# Functional neurological signs in hypermobile Ehlers–Danlos syndrome and hypermobile spectrum disorders with suspected neuropathic pain

**DOI:** 10.1002/brb3.3441

**Published:** 2024-02-26

**Authors:** Aurore Fernandez, Manon Jaquet, Bérengère Aubry‐Rozier, Marc Suter, Selma Aybek, Chantal Berna

**Affiliations:** ^1^ Center for Integrative and Complementary Medicine, Department of Anesthesiology Lausanne University Hospital (CHUV) Lausanne Switzerland; ^2^ Pain Center, Department of Anesthesiology Lausanne University Hospital (CHUV) Lausanne Switzerland; ^3^ Faculty of Biology and Medicine University of Lausanne Lausanne Switzerland; ^4^ The Sense Innovation and Research Center Lausanne and Sion Switzerland; ^5^ Department of Rheumatology Lausanne University Hospital (CHUV) Lausanne Switzerland; ^6^ Neurology, Faculty of Science and Medicine Fribourg University Fribourg Switzerland

**Keywords:** chronic pain, functional neurological disorder, functional neurological positive signs, hypermobile Ehlers–Danlos syndrome, hypermobile spectrum disorder

## Abstract

**Background:**

The hypermobile Ehlers–Danlos syndrome (hEDS) and hypermobility spectrum disorders (HSD) are connective tissue disorders characterized by generalized joint hypermobility, associated with chronic pain and several symptoms, such as fatigue, dysautonomia, as well as psychiatric co‐morbidities. Clinical observations of unusual manifestations during systematic sensory testing raised the question of a possible co‐existence with a functional neurological disorder (FND). Hence, this study aimed to assess the presence of positive functional neurological signs (FNS) in a cohort of patients with hEDS/HSD.

**Methods:**

The clinical data of hEDS/HSD patients (*N* = 24) were retrospectively analyzed and compared to a prospectively recruited age‐/sex‐matched healthy control group (*N* = 22). Four motor‐ and three sensory‐positive FNS were assessed.

**Results:**

Twenty‐two patients (92%) presented at least one motor or sensory FNS. Five patients (21%) presented only a single FNS, 14 presented between 2 and 4 FNS (58%), and 3 patients presented 5 or more FNS (12%). None of the healthy controls presented motor FNS, and only two presented a sensory FNS.

**Conclusions:**

The presence of FNS in hEDS/HSD deserves better clinical detection and formal diagnosis of FND to offer more adequate care in co‐morbid situations. In fact, FND can severely interfere with rehabilitation efforts in hEDS/HSD, and FND‐targeted physical therapy should perhaps be combined with EDS/HSD‐specific approaches.

## Graphical abstract

This paper aimed to assess the presence of positive functional neurological signs (FNS) in a cohort of patients with hEDS/HSD. This figure shows the number of FNS and the coexistence between sensory (blue) and motor (red) FNS.

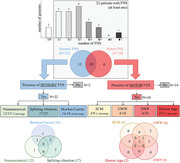

## 1 INTRODUCTION

Hypermobile Ehlers–Danlos syndrome (hEDS) is a heritable connective tissue disorder. The diagnosis relies on clinical symptoms, including generalized joint hypermobility (GJH), systemic manifestations of connective tissue disorder, and musculoskeletal complications (Malfait et al., 2017). Patients suffering from symptomatic GJH (joint subluxations, dislocations, sprains, and other injuries), yet not fulfilling the criteria for hEDS, are diagnosed with hypermobility spectrum disorder (HSD) (Castori et al., 2017). Associated symptoms such as fatigue and dysautonomia (Chopra et al., 2017), as well as psychiatric and psychological co‐morbidities (Bulbena et al., 2017), are described in hEDS/HSD. Pain is a prominent feature, with diffuse complaints (musculoskeletal, gastro‐intestinal, etc.), which can be attributed to a combination of nociceptive, nociplastic, and neuropathic pain (Fernandez et al., 2022). In fact, we demonstrated a frequent neuropathic component with evidence of small fiber neuropathy (SFN) in a cohort of hEDS/HSD patients (*N* = 79) (Fernandez et al., 2022). The small‐fiber evaluation relied on skin biopsies and quantitative sensory testing (QST). During this sensory assessment, we observed unusual reports, such as migratory pain, nonanatomical distribution of pain, and lingering after‐sensations following touch. These features, not explained by the SFN, could hint to a co‐existence with a sensory functional neurological disorder (FND). Previously a diagnosis of exclusion, FND is now a rule‐in diagnosis with specific positive signs (Aybek & Perez, 2022). Given there is to date no characterization of FND in patients suffering from hEDS/HSD, a first step could be to describe the distribution of positive functional neurological signs (FNS). Hence, in this pilot study, we aimed to describe the presence of such FNS in a cohort of patients with hEDS/HSD.

## 2 METHODS

### 2.1 Study population

This study is a retrospective analysis of clinical data from adult patients suffering from hEDS/HSD diagnosed according to the 2017 criteria (Malfait et al., 2017) by a rheumatologist, who were referred to an academic pain center based on the presence of chronic pain with potential neuropathic symptoms or dysautonomia. The patient cohort is a subset of previously described population (Aubry‐Rozier et al., 2021; Fernandez et al., 2022) who underwent a neurological assessment (between 09‐2019 and 10‐2020; CERVD 2019‐00093). A healthy control group was prospectively recruited, matching mean patient age and sex (CERVD 2020‐02259) while excluding chronic pain or any conditions causing neuropathy.

### 2.2 Functional neurological signs (FNS)

Three positive *motor* FNS were assessed in all patients (Aybek & Perez, 2022): sternocleidomastoid muscle strength asymmetry, give‐way weakness (not due to pain), and drift without pronation. A fourth one, the Hoover sign, was tested only in patients reporting unilateral limb weakness.

Three positive *sensory* FNS were assessed: splitting of vibration sense (measured on the forehead and the sternum), nonanatomical distribution of tactile detection, and the Bowlus–Currier test (i.e., putting the hands in a position that creates confusion regarding left/right location). More details on the choice of these FNS and the assessment procedures are provided in the Supporting Information section.

### 2.3 Clinical data and analysis

Patients filled out questionnaires assessing pain characteristics, impact on daily life, and psychological health (see the Supporting Information section). An SFN was considered “definite” if both the QST and the skin biopsy were abnormal, “possible” if either one or the other was abnormal, and “excluded” if both were normal (Fernandez et al., 2022). Exploratory analyses were performed to evaluate links between FNS and symptoms severity. A non‐parametric Kruskal–Wallis test compared the number of positive FNS depending on the likelihood of SFN (definite, possible, or excluded). A median split was performed on the number of positive FNS (median = 3 FNS), comparing pain intensity and interference between those with little (<3) and many (≥3).

## 3 RESULTS

### 3.1 Study participants

The sample was composed of 24 patients, 14 hEDS and 10 HSD (42%), 22 women (92%) aged from 21 to 54 (see Table 1). The healthy controls did not differ in terms of age and sex. All patients reported chronic pain (BPI‐PS 5.9 ± 2.0) interfering with their daily life (BPI‐PI 5.5, SD = 2.4), with 75% reporting neuropathic characteristics (DN4+), 54% categorized with definite SFN, and 29% with possible SFN (details in the Supporting Information section).

**TABLE 1:** Demographic characteristics and symptoms description of the study population.

	hEDS/HSD patients (*N* = 24)	Controls (*N* = 22)
Female (%)	22 (92%)	20 (91%)
Age *(mean ± SD)*	37.0 ± 10.6	38.9 ± 11.3
BPI pain severity *(mean ± SD)*	5.9 ± 2.0	–
BPI pain interference *(mean ± SD)*	5.5 ± 2.4	–
Kinesiophobia (mean ± SD) *% of positivity (≥40)*	40.2 ± 11.0 *65%*	–
Pain catastrophizing (mean ± SD) *% of positivity (≥20)*	23.0 ± 10.8 *52%*	–
Anxiety (mean ± SD) *% of positivity (≥8)*	10.6 ± 3.1 *91%*	–
Depression (mean ± SD) *% of positivity (≥8)*	7.7 ± 4.0 *52%*	–
QOL physical health *(score/100, mean ± SD)*	29.2 ± 10.1	–
QOL psychological health *(score/100, mean ± SD)*	52.3 ± 17.4	–
QOL social relationships *(score/100, mean ± SD)*	55.7 ± 17.5	–
QOL environment *(score/100, mean ± SD)*	61.4 ± 16.7	–
SFN‐SIQ *(score/100, mean ± SD)*	21.1 ± 8.7	–
DN4 (mean ± SD) *% of positivity (≥4)*	4.6 ± 2.1 *75%*	–

*Note*: Data are presented as mean, standard deviation (SD), and percentage of positivity = *N* reaching clinically validated cut‐off of scores (considered cut‐off).

Abbreviations: BPI, brief pain inventory; DN4, douleur neuropathique 4; HADS, hospital anxiety and depression scale; hEDS, hypermobile Ehlers–Danlos syndrome; HSD, hypermobility spectrum disorders; QOL, quality of life from WHO‐bref; SFN‐SIQ, small‐fiber neuropathy symptoms inventory questionnaire.

### 3.2 Functional neurological signs (FNS)

**FIGURE 1:**
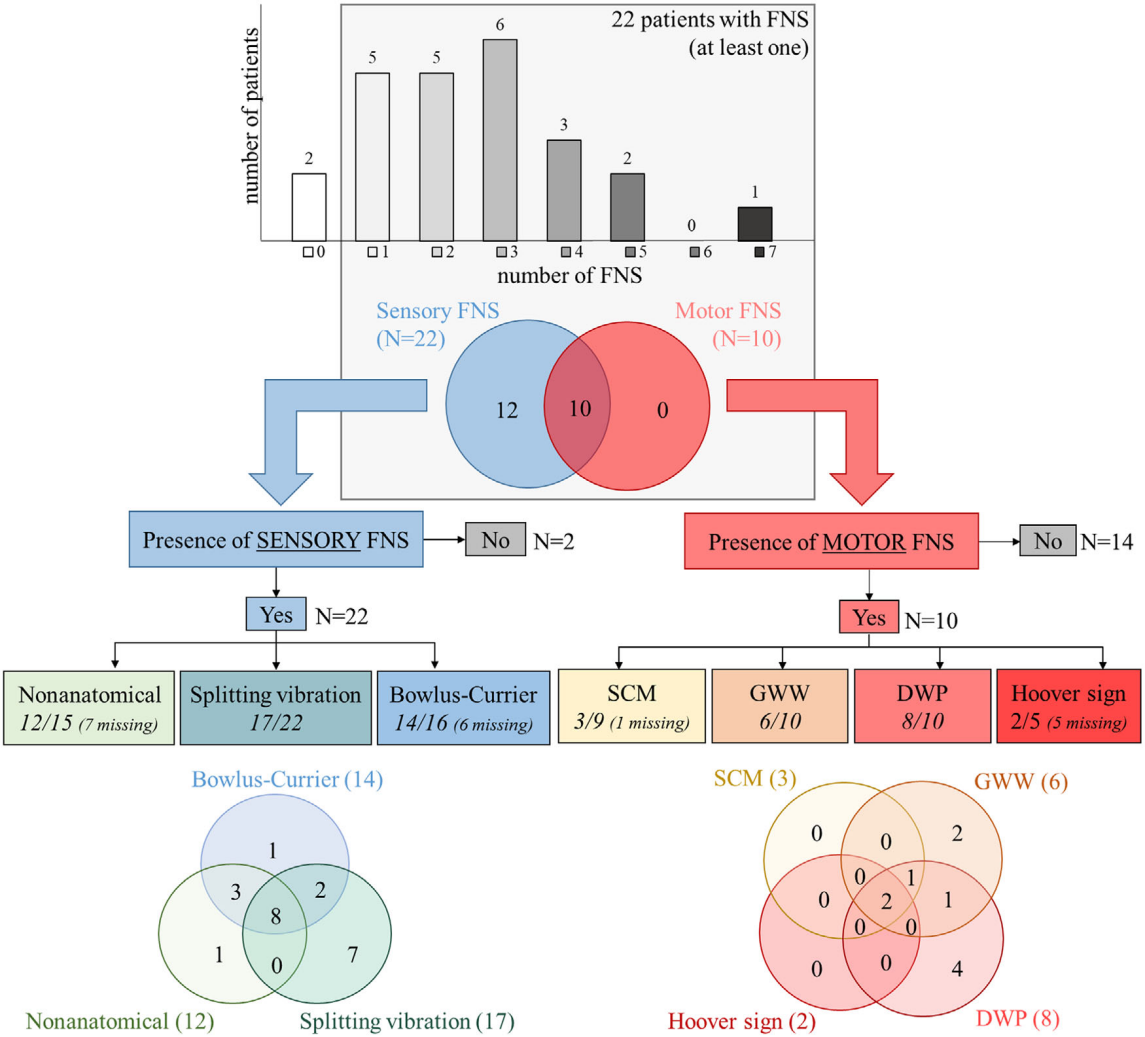
Illustration of the presence of positive functional neurological signs (FNS) in the hypermobile Ehlers–Danlos syndrome (hEDS)/hypermobility spectrum disorder (HSD) patient population (N= 24). At the top, the full population is depicted, illustrating the number of positive FNS in the sample. Then, the coexistence between sensory (blue) and motor (red) signs is represented in a Venn diagram. At the bottom, the number of patients with each of the signs, either isolated or in combination with others (at the intersections), are presented for sensory FNS on the left and for motor FNS on the right. DWP, drift without pronation; GWW, give‐way weakness; SCM, sternocleidomastoid sign.

Twenty‐two patients out of 24 (92%) presented at least 1 motor or sensory FNS (Figure 1). Five patients (21%) presented only a single FNS, 14 presented between 2 and 4 FNS (58%), and 3 patients presented 5 or more FNS (12%). None of the healthy controls presented motor FNS, and only two presented a sensory FNS (splitting of the vibration sense). The proportion of controls presenting this sensory FNS is significantly lower than in patients (*X* ^2^(1, *N* = 46) = 18.0, *p* < .001). Most of the hEDS/HSD patients presented sensory FNS, either isolated (pure sensory FNS: *N* = 12) or in combination with motor FNS (mixed sensory and motor FNS, *N* = 10). No patient presented isolated motor FNS (Figure 1).

### 3.3 Association between FNS and other symptoms

There was no significant difference in the number of FNS between the SFN likelihood groups (definite, possible, and excluded) (H(2) = 5.7, *p* = .06). The patients with several FNS (≥3; *N* = 12) reported significantly higher pain interference with daily life (6.8 ± 2.0 vs. 4.6 ± 2.2; *t*(22) = 2.4, *p* = .02) and higher pain intensity (trend‐level 6.7 ± 1.8 vs. 5.1 ± 1.7; *t*(22) = 2.0, *p* = .05) than those with few (<3; *N* = 12).

## 4 DISCUSSION

The purpose of this descriptive study was to assess the presence of positive FNS in patients diagnosed with hEDS/HSD compared to a control group. We observed more frequent positive FNS in hEDS/HSD patients (92%, *N* = 24, sensory > motor) than in healthy controls. To our knowledge, this is the first study focusing on the detection of positive functional neurological disorder signs in patients with an hEDS/HSD diagnosis. A causal link cannot be established due to the limited sample size and retrospective design. Nevertheless, this co‐morbidity deserves further research.

FND is frequent in chronic pain with a prevalence of 17%, according to a recent report (Mason et al., 2023). Despite not having formally established an FND diagnosis in our study, the co‐occurrence of FNS seems even more frequent in hEDS/HSD . Further work, including a formal FND diagnosis, is now required. Yet, in our cohort, it was not possible to determine if the FNS were due to joint hypermobility, neuropathic or chronic pain more broadly, or even common psychological co‐morbidities/predisposing factors. This was, however, not the point of this descriptive report.

There is an emerging interest in a potential co‐morbidity between hEDS/HSD and FND. Previous studies came from the reverse perspective: they examined patients with diagnosed FNDs, testing for GJH, but without a formal diagnosis of hEDS/HSD. GJH was assessed with the Beighton scale in patients with functional seizures (*N* = 42, positive in 57%) (Koreki et al., 2022) and in adolescents with digestive functional syndromes (*N* = 45; positive in 56%) (Kovacic et al., 2014). Hypermobility self‐reports were collected in a mixed sample of FNDs (*N* = 20, positive GJH in 55%) (Nistico et al., 2022) or in fixed dystonia (*N* = 28, positive in 32%) (Kassavetis et al., 2012). Retrospective reviews of FND patients’ clinical notes revealed mentions of GJH (*N* = 100, positive in 21%) (Delgado et al., 2022) or mentions of EDS diagnosis (*N* = 190, positive in 8.4%) (Margolesky et al., 2022). In the general adult population, GJH prevalence ranges between 10% and 20% (Nicholson et al., 2022), hence the prevalence appearst o be higher in most of these FND samples.

Our preliminary and descriptive study does not allow to establish any causal links between hEDS/HSD and the presence of FNS. Nevertheless, conceptually, several hypotheses could be formulated. Aberrant interoceptive signals and altered autonomic control, described in hEDS/HSD patients, may contribute to developing FND (Aubry‐Rozier et al., 2021; Fernandez et al., 2022). The current neurobiological framework for FND is based on a model of hierarchical Bayesian inference in the brain in terms of perception and action arising based on prior beliefs and sensory information (Edwards et al., 2012). Deficits of the somatosensory system (Fernandez et al., 2022), with resulting sensory alterations, could lead to misguided priors, hence starting erroneous feedback loops from youth on (Perez et al., 2021). Additionally, physical injuries (repeatedly observed in hEDS/HSD) have been reported as favoring the development of functional complaints of weakness (Stone et al., 2012). Finally, kinesiophobia, frequently described in hEDS/HSD, could lead to movement avoidance or limb immobilization, known to contribute to fixed dystonia (Schrag et al., 2004) and frequently observed in FND in general.

Even though this research is promising, there are some limitations. First, the small sample size calls for validations in larger populations, including hEDS/HSD without neuropathic pain symptoms. In fact, the tested sample was not fully representative of the larger hEDS/HSD population, as it selected people reporting neuropathic pain symptoms (representing 50% of the larger cohort [Fernandez et al., 2022]). Furthermore, no FND diagnosis was established. There is to date no validated cut‐off for the number of FNS needed to establish an FND diagnosis, which usually relies on an expert neurological exam. Most of the chosen FNS had been validated in FND (see the Supporting Information section) but not in chronic pain populations. Non‐dermatomal sensory loss has a prevalence of 25%–50% in chronic pain (Mailis‐Gagnon & Nicholson, 2011), but the specificity of the presence of such a sign has not yet been evaluated. Further work could allow to decide on an optimal battery of tests in the hEDS/HSD population and involve a neurological evaluation to provide a diagnosis. Nevertheless, the previously described somatic sensory alterations in hEDS/HSD could affect the specificity and sensitivity of the FNS tests, especially sensory ones (Fernandez et al., 2022). Moreover, some experts in the field would argue that functional sensory signs are not that reliable (Stone & Vermeulen, 2016). Proper controls of patients with either chronic pain and/or small fiber‐related sensory alterations should be considered for future studies.

In conclusion, extensive characterization is still required to better understand the link among hEDS/HSD, FND, and pain. Given the frequency of FNS in patients with hEDS/HSD, this association deserves more systematic detection and, if positive, evaluation for FND diagnosis to offer adequate care in co‐morbid situations. In fact, FND can severely interfere with rehabilitation efforts in hEDS/HSD, and FND‐targeted physical therapy (Perez et al., 2021) should perhaps be combined with EDS/HSD‐specific approaches.

## AUTHOR CONTRIBUTIONS

**Aurore Fernandez**: Conceptualization; investigation; writing—original draft; methodology; validation; writing—review and editing; formal analysis; data curation; project administration. **Manon Jaquet**: Funding acquisition; writing—review and editing; methodology; investigation; data curation; project administration. **Bérengère Aubry‐Rozier**: Conceptualization; methodology; validation; writing—review and editing; supervision. **Marc Suter**: Conceptualization; methodology; validation; writing—review and editing; supervision. **Selma Aybek**: Funding acquisition; Conceptualization; methodology; validation; visualization; formal analysis; supervision. **Chantal Berna**: Conceptualization; methodology; validation; writing—review and editing; supervision; resources; data curation; project administration; formal analysis.

### PEER REVIEW

The peer review history for this article is available at https://publons.com/publon/10.1002/brb3.3441.

## Supporting information

Supporting Information.

## Data Availability

The data that support the findings of this study are available in the Supporting Information section of this article.
