# Supplementary material for: Functional neurological signs in hypermobile Ehlers–Danlos syndrome and hypermobile spectrum disorders with suspected neuropathic pain
Source: Brain Behav. 2024 Feb 26;14(2):e3441. doi: 10.1002/brb3.3441 (PMC10897362; doi:10.1002/brb3.3441)
Supplement: Supplementary file 1 — Supporting Information. [file BRB3-14-e3441-s001.docx]

***Supplementary methods:***

## Functional neurological signs (FNS)

Four positive motor FNS were assessed: we selected 4 motor signs that have good inter-rater reliability [1] and that have been validated in at least 2 studies: the sterno-cleido-mastoid muscle strength asymmetry, give-way weakness and drift without pronation. If subjective lower limb weakness was reported, the Hoover sign was included.


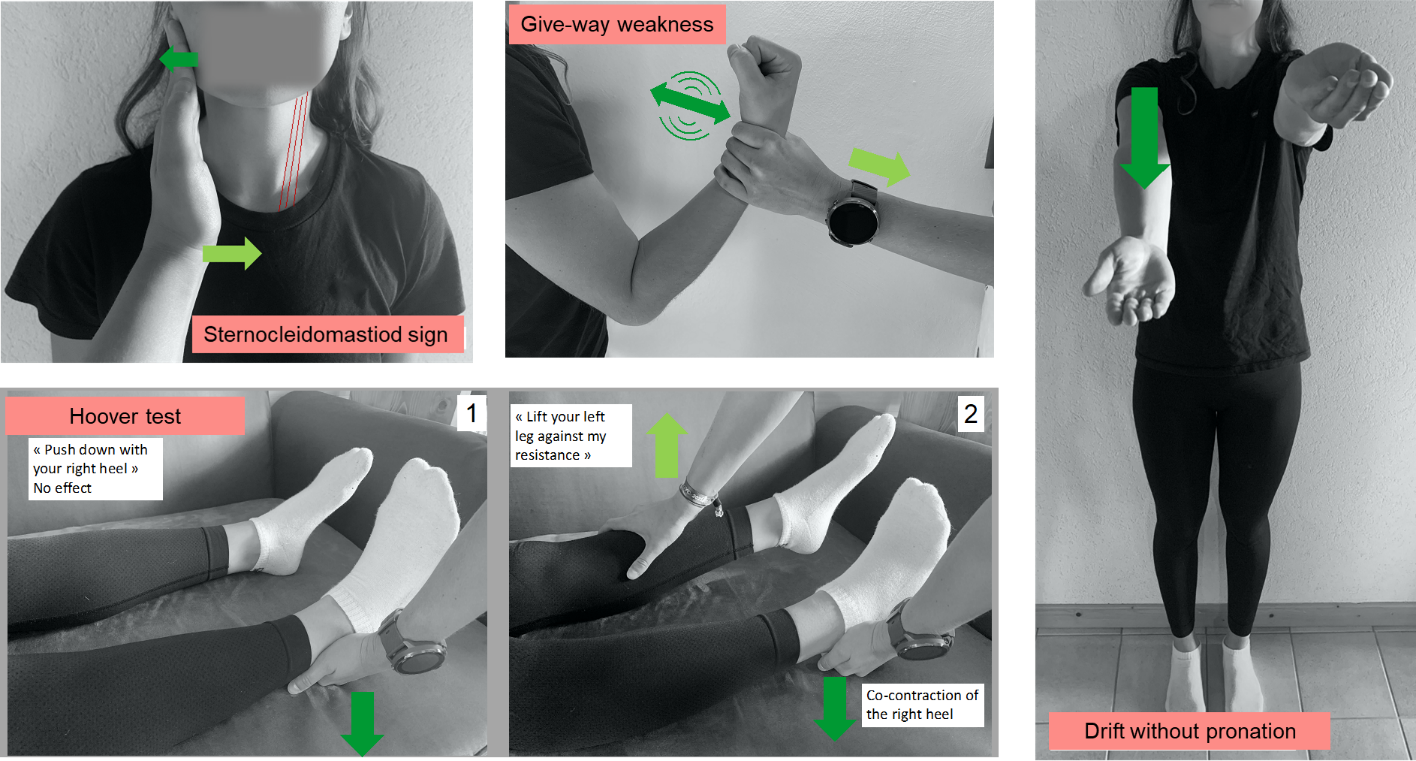


The Sternocleidomastoid sign tests was evaluated in a sitting position. The participant was asked to turn his head toward each side against the examiner’s resistance. The SCM sign was considered a positive sign if a the side of weakness was identical as the reported weakness side recorded since a leftward weakness should be reflected by right SCM weakness and vice versa [2]. This sign was validated in 2 studies in patients with mixed sensorimotor or pure motor FND (pooled N=50 FNDs patients vs N=50 controls with neurological organic condition)[2, 3]. This test had a pooled specificity of 93%, a sensitivity of 53% and an excellent inter-rater reliability (κ 0.83) [1].

The Give-way weakness sign was considered positive when patients can initially produce a normal strength, but when tested against the examiner’s resistance, the limb suddenly collapses as if it “gives way” [4]. Strength was evaluated for head rotation, arm abduction, elbow flexion, wrist extension, hip flexion, knee extension, dorsiflexion of the foot. This test was validated in 3 studies in patients with functional weakness N=107 [5]; mixed sensorimotor or pure motor FND N=20 [3, 6] with a pooled specificity of 97%, a sensitivity of 67%, and a good inter-rater reliability (κ 0.6) [1].

The drift without pronation is tested with the patient standing with his arms held up in a supinated position (palms facing upwards, fingers adducted) and eyes closed. This FNS is considered positive if a downward arm drift occur without pronation of the hand [7]. This test was validated in 2 studies [3, 7] with a pooled specificity of 96%, a sensitivity of 78% and a good inter-rater reliability (κ 0.78) [1].

The Hoover sign is tested with the patient lying down on his back and considered positive when there is weakness of voluntary hip extension in the presence of normal involuntary hip extension during contralateral hip flexion against resistance [8]. This test is performed only is a lower limb weakness is present. This test was validated in 5 studies [3, 5, 9-11] with a pooled specificity of 99.5% and a sensitivity of 61% [1].

Three positive sensory FNS were assessed: we selected two signs that were validated and deemed reliable [3] (splitting of vibration sense; non-anatomical distribution of tactile detection) and as we were interested in incongruences in responses to sensory testing in the population we added the Bowlus-Currier test even if it has only been evaluated in one single previous study [12].


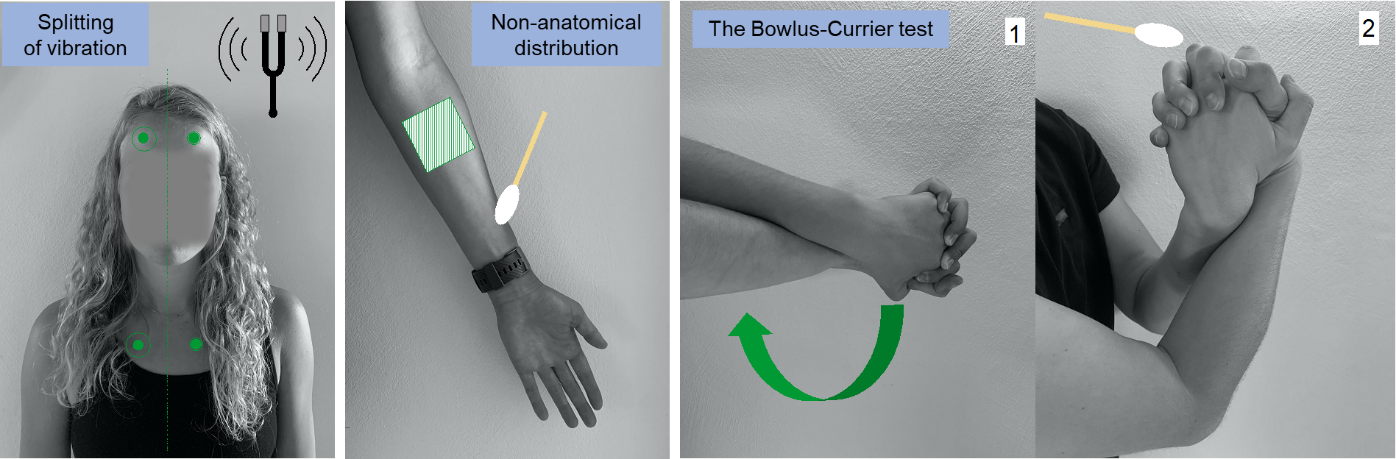


The Splitting of vibration sensation is considered positive if there is a right/left difference

in pallesthesia using tuning fork placed across the forehead or sternum. The sensation should be identical since the same bone is involved [3]. This sign was validated in 4 studies and showed a good interrater reliability (κ 0.68) with an 88% specificity [3].

The Non-anatomical distribution is defined by a diminished or abnormal sensation (such as hypoesthesia, rough or burning sensation) which does not fit a dermatomal pattern [13]. Tactile detection was assessed using a cotton wisp applied on the main dermatomes. Each hypo/hyper sensation was reported by the examiner on a map of a human body. This mapping was then blindly evaluated by a trained neurologist (S.A.) to judge anatomical coherence (e.g pattern of symmetric sensory loss suggesting a polyneuropathy, dermatome etc.). This sign was validated in 2 studies and showed a fair interrater reliability (κ 0.23) with a 90% specificity [3].

The Bowlus-Currier test relies on putting the hands in a position that creates a confusion regarding left/right location [13]. The sensory reports of perception from a cotton wisp touch in the “mixed-up” position are compared with the previous assessment of fingers sensitivity, with discrepancies between both tests being reported as abnormal. This sign was not formally validated in a controlled study.

## Questionnaires

Pain intensity and interference with daily life were assessed with the Brief Pain Inventory (BPI) [14]. Neuropathic characteristics evaluated with the Douleur Neuropathique 4 questionnaire (DN4) [15]. We evaluated the psychological health using the Hospital Anxiety and Depression Scale (HADS) [16], pain coping through the Pain Catastrophizing Scale (PCS) [17], and fear of movement through the TAMPA scale of kinesiophobia (TSK) [18]. Quality of life was assessed through the World Health Organization Quality of Life BREF questionnaire (WHO-QOL BREF) [19]. Raw scores were converted into the transformed domains scores ranging from 0 to 100 for physical health, psychological domain, social relationships and environment.

***Supplementary results:***

| Supplementary Table 1. Demographic characteristics and symptoms description of the study population | | |
| --- | --- | --- |
|  | hEDS/HSD Patients  *(N=24)* | Controls  *(N=22)* |
| Female  (%) | 22 (92%) | 20 (91%) |
| Age  *(mean ± SD)* | 37.0 ± 10.6 | 38.9 ± 11.3 |
| BPI Pain Severity  *(mean ± SD)* | 5.9 ± 2.0 | - |
| BPI Pain Interference  *(mean ± SD)* | 5.5 ± 2.4 | - |
| Kinesiophobia (mean ± SD)  *% of positivity (≥40)* | 40.2 ± 11.0  *65 %* | - |
| Pain catastrophizing (mean ± SD)  *% of positivity (≥20)* | 23.0 ± 10.8  *52 %* | - |
| Anxiety(mean ± SD)  *% of positivity (≥8)* | 10.6 ± 3.1  *91 %* | - |
| Depression (mean ± SD)  *% of positivity (≥8)* | 7.7 ± 4.0  *52 %* | - |
| QOL physical health  *(score /100, mean ± SD)* | 29.2 ± 10.1 | - |
| QOL psychological health  *(score /100, mean ± SD)* | 52.3 ± 17.4 | - |
| QOL social relationships  *(score /100, mean ± SD)* | 55.7 ± 17.5 | - |
| QOL environment  *(score /100, mean ± SD)* | 61.4 ± 16.7 | - |
| SFN-SIQ  *(score /100, mean ± SD)* | 21.1 ± 8.7 | - |
| DN4 (mean ± SD)  *% of positivity (≥4)* | 4.6 ± 2.1  *75 %* | - |
| Data is presented as mean, standard deviation (SD) and percentage of positivity=N reaching clinically validated cut-off of scores (considered cut-off). BPI: Brief Pain Inventory; SFN-SIQ: Small Fiber Neuropathy Symptoms Inventory Questionnaire; QOL: Quality of Life from WHO-Bref; DN4: Douleur Neuropathique 4; HADS: Hospital Anxiety and Depression Scale. | | |

Supplementary table 2: This supplementary table displays the raw data for functional neurological signs, small fiber neuropathy assessment, questionnaires and diagnostic criteria

SCM: sterno cleido-mastoid sign; DWP: drift without pronation; BC: Bowlus-Currier; Non-anato: The Non-anatomical distribution; Vibration: Splitting of vibration sensation; IENFD: intraepidermal nerve fiber density; QST: quantitative sensory testing; BPI: Brief Pain Inventory (PS: pain severity; I: interference with daily life); DN4: Douleur Neuropathique 4 questionnaire; HADS: Hospital Anxiety and Depression Scale (A: anxiety; D: depression); PCS: Pain Catastrophizing Scale; TSK: Tampa scale of kinesiophobia; QOL: World Health Organization Quality of Life BREF questionnaire; HV: healthy volunteer; diag: diagnostic; 1: CRITERION 1 – Generalized Joint Hypermobility= Beighton scale ; 2A: more than 5/12 systemic manifestations; 2B: positive family history; 2C: At least 1 musculoskeletal complication; 3: criterion 3 filled. See details of diagnostic criteria below or <https://www.ehlers-danlos.com/wp-content/uploads/2017/05/hEDS-Dx-Criteria-checklist-1.pdf>

*
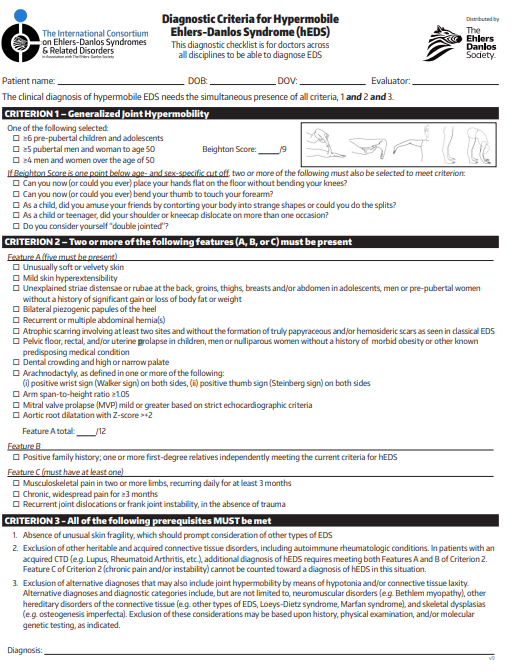
*

References

1. Aybek, S. and D.L. Perez, Diagnosis and management of functional neurological disorder. BMJ, 2022. 376: p. o64.

2. Horn, D., et al., Testing Head Rotation and Flexion Is Useful in Functional Limb Weakness. Mov Disord Clin Pract, 2017. 4(4): p. 597-602.

3. Daum, C., et al., Interobserver agreement and validity of bedside 'positive signs' for functional weakness, sensory and gait disorders in conversion disorder: a pilot study. J Neurol Neurosurg Psychiatry, 2015. 86(4): p. 425-30.

4. Aboud, O., et al., Positive clinical signs in neurological diseases - An observational study. J Clin Neurosci, 2019. 59: p. 141-145.

5. Stone, J., C. Warlow, and M. Sharpe, The symptom of functional weakness: a controlled study of 107 patients. Brain, 2010. 133(Pt 5): p. 1537-51.

6. Chabrol, H., G. Peresson, and M. Clanet, Lack of specificity of the traditional criteria for conversion disorders. Eur Psychiatry, 1995. 10(6): p. 317-9.

7. Daum, C. and S. Aybek, Validity of the "Drift without pronation" sign in conversion disorder. BMC Neurol, 2013. 13: p. 31.

8. Stone, J., A. Carson, and M. Sharpe, Functional symptoms and signs in neurology: assessment and diagnosis. J Neurol Neurosurg Psychiatry, 2005. 76 Suppl 1: p. i2-12.

9. Sonoo, M., Abductor sign: a reliable new sign to detect unilateral non-organic paresis of the lower limb. J Neurol Neurosurg Psychiatry, 2004. 75(1): p. 121-5.

10. Tinazzi, M., et al., Abduction finger sign: a new sign to detect unilateral functional paralysis of the upper limb. Mov Disord, 2008. 23(16): p. 2415-9.

11. McWhirter, L., et al., Hoover's sign for the diagnosis of functional weakness: a prospective unblinded cohort study in patients with suspected stroke. J Psychosom Res, 2011. 71(6): p. 384-6.

12. Bowlus, W.E. and R.D. Currier, A Test for Hysterical Hemianalgesia. N Engl J Med, 1963. 269: p. 1253-4.

13. Daum, C., M. Hubschmid, and S. Aybek, The value of 'positive' clinical signs for weakness, sensory and gait disorders in conversion disorder: a systematic and narrative review. J Neurol Neurosurg Psychiatry, 2014. 85(2): p. 180-90.

14. Tan, G., et al., Validation of the Brief Pain Inventory for chronic nonmalignant pain. J Pain, 2004. 5(2): p. 133-7.

15. Bouhassira, D., et al., Comparison of pain syndromes associated with nervous or somatic lesions and development of a new neuropathic pain diagnostic questionnaire (DN4). Pain, 2005. 114(1-2): p. 29-36.

16. Zigmond, A.S. and R.P. Snaith, The hospital anxiety and depression scale. Acta Psychiatr Scand, 1983. 67(6): p. 361-70.

17. Sullivan, M.J., S.R. Bishop, and J. Pivik, The Pain Catastrophizing Scale: Development and validation. Psychological Assessment, 1995. 7(4): p. 524-532.

18. French, D.J., et al., Fear of movement/(re)injury in chronic pain: a psychometric assessment of the original English version of the Tampa scale for kinesiophobia (TSK). Pain, 2007. 127(1-2): p. 42-51.

19. Development of the World Health Organization WHOQOL-BREF quality of life assessment. The WHOQOL Group. Psychol Med, 1998. 28(3): p. 551-8.
